# New Methacrylated Biopolymer-Based Hydrogels as Localized Drug Delivery Systems in Skin Cancer Therapy

**DOI:** 10.3390/gels9050371

**Published:** 2023-05-01

**Authors:** Andreea Luca, Isabella Nacu, Sabina Tanasache, Cătălina Anişoara Peptu, Maria Butnaru, Liliana Verestiuc

**Affiliations:** 1 Department of Biomedical Sciences, Faculty of Medical Bioengineering, “Grigore T. Popa” University of Medicine and Pharmacy, 700115 Iasi, Romania; andreea.luca@umfiasi.ro (A.L.); nacu.isabella@gmail.com (I.N.); tanasachesabina@gmail.com (S.T.); maria.butnaru@umfiasi.ro (M.B.); 2 “Petru Poni” Institute of Macromolecular Chemistry, 700487 Iasi, Romania; 3 Cristofor Simionescu Faculty of Chemical Engineering and Environmental Protection, Gheorghe Asachi Technical University of Iaşi, 700050 Iasi, Romania; catipeptu@yahoo.co.uk

**Keywords:** hydrogels, biopolymers, poly(acrylamide), Doxorubicin, skin cancer therapy

## Abstract

The aim of the present work was to obtain drug-loaded hydrogels based on combinations of dextran, chitosan/gelatin/xanthan, and poly (acrylamide) as a sustained and controlled release vehicle of Doxorubicin, a drug used in skin cancer therapy that is associated with severe side effects. Hydrogels for use as 3D hydrophilic networks with good manipulation characteristics were produced using methacrylated biopolymer derivatives and the methacrylate group’s polymerization with synthetic monomers in the presence of a photo-initiator, under UV light stimulation (365 nm). Transformed infrared spectroscopy analysis (FT-IR) confirmed the hydrogels’ network structure (natural–synthetic composition and photocrosslinking), while scanning electron microscopy (SEM) analysis confirmed the microporous morphology. The hydrogels are swellable in simulated biological fluids and the material’s morphology regulates the swelling properties: the maximum swelling degree was obtained for dextran–chitosan-based hydrogels because of their higher porosity and pore distribution. The hydrogels are bioadhesive on a biological simulating membrane, and values for the force of detachment and work of adhesion are recommended for applications on skin tissue. The Doxorubicin was loaded into the hydrogels and the drug was released by diffusion for all the resulting hydrogels, with small contributions from the hydrogel networks’ relaxation. Doxorubicin-loaded hydrogels are efficient on keratinocytes tumor cells, the sustained released drug interrupting the cells’ division and inducing cell apoptosis; we recommend the obtained materials for the topical treatment of cutaneous squamous cell carcinoma.

## 1. Introduction

In recent decades, skin cancer has become one of the most prevalent types of cancer, generating increased expenses for healthcare systems and representing a burden for the medical specialists and the affected patients [1,2]. The statistics from World Health Organization show that skin cancer is the fifth most reported type of cancer in the world [3], and its severity depends on the type of cells implicated [4]. Non-melanoma skin cancer represents approximately 95% of the total number of cases, but 80% of the deaths caused by skin cancer are attributed to the melanoma type, with a 5-year survival rate of only 30% for the advanced stage [3]. It is estimated that, by 2040, increases of 50% in the number of melanoma cases and 68% in deaths caused by melanoma will be registered [5]. Treating cancer is a continuous challenge, as the ideal type of therapeutic procedure/treatment has not yet been found. Systemic therapy, such as chemotherapy or immunotherapy, cannot differentiate between cancer and normal cells. The side-effects of systemic therapy are a consequence of the modifications in the metabolite profile given by phenotypic and cell-function alterations, making targeted drug delivery an important treatment option, especially for cancers that affect an exposed organ, such as skin cancer [6,7]. As such, several strategies for targeted drug delivery have been developed as an alternative to systemic therapy or surgical tumor removal. Therefore, topical chemotherapeutic and immunotherapeutic agents released from different bioengineered structures have been tested, some of them with promising results [8,9,10].

Hydrogels represent extremely versatile biomaterials with wide applications in tissue engineering and drug delivery; they can be used in an injectable form [11] or as transdermal patches [12,13], nanoparticles [14,15,16], etc. They are 3D networked materials and offer the possibility of the local delivery of increased concentrations of anti-tumor drugs, and their release profile can be controlled by the composition and processing conditions [17].

Natural and synthetic polymer-based hydrogels offer the advantages of both classes of biomaterials: the biocompatibility of the natural polymers, along with the structural versatility of the synthetic classes. Polysaccharides possess properties that make them suitable drug delivery systems, especially their biocompatibility, biodegradability, and hydrophilicity, and the possibility of modifying them with different categories of molecules, biological compounds, and moieties. Chitosan is a biocompatible polysaccharide with good availability and a chemically versatile structure, especially due to the presence of hydroxyl and amino groups [18]. This cationic polysaccharide has attracted particular attention as a trigger for drugs in cancer therapy because of its electrostatic interactions with tumor cells that appear to disrupt the membrane’s integrity, and because it can induce cell apoptosis as it determines the accumulation of high levels of reactive oxygen species [19,20,21,22].

Polysaccharides such as dextran or xanthan possess properties that offer promising results in cancer therapy, in which macromolecules can be used either unmodified or after chemical modifications [23,24,25]. Dextran has been used as a drug carrier through the cell membrane, especially for polyphenols, due to its increased targeting of the tumor tissues [26]. Meanwhile, xanthan was tested in tissue engineering applications and cancer therapy as a thermoreversible hydrogel in combinations with alginate or chitosan, based on polymers’ synergistic interactions in an aqueous medium [27,28,29].

Gelatin’s structural modifications have been studied in particular depth due to the presence of amino acid side chains on the macromolecule. Gelatin methacryloyl (GelMA) was used in different forms, as a 3D scaffold, injectable hydrogels, bioink for 3D printing, or as electrospun fibers. The chemical modification offers a cytocompatible gelatin crosslinking technique, which stabilizes the bioengineered structures at physiologic temperatures [30,31,32,33].

In this study, methacrylated natural polymers (chitosan, dextran, xanthan, and gelatin) were crosslinked with synthetic acrylamide and N,N’-methylenebis(acrylamide) to obtain new hydrogels for drug delivery systems in skin cancer therapy. Chitosan, gelatin, dextran, and xanthan were modified through graft polymerization with methacrylic anhydride. The chemical modification of the polysaccharides improved both their behavior when participating in the crosslinking reaction and the biocompatibility of the final mixture. The obtained hydrogels were used as drug carriers for doxorubicin, an anti-tumor agent that prevents DNA replication and affects the activity of topoisomerase II and cell functioning by binding to the cell membrane [34]. Dextran increases the stability of doxorubicin [35]; therefore, it was chosen as a constant structural component that was present in each of the obtained hydrogels. The cytotoxicity of the hydrogels and their behavior as anti-tumor drug carriers were tested on an A431 squamous carcinoma cell line. The originality of this study constitutes the mixture of different methacrylated polysaccharides with synthetic polymers, acrylamide, and bis-acrylamide to obtain new hydrogels with structures that allow for adequate drug release kinetics, for use in cutaneous squamous cell carcinoma therapy.

## 2. Results and Discussion

### 2.1. Synthesis of Hydrogels and FT-IR Data

**Figure 1:**
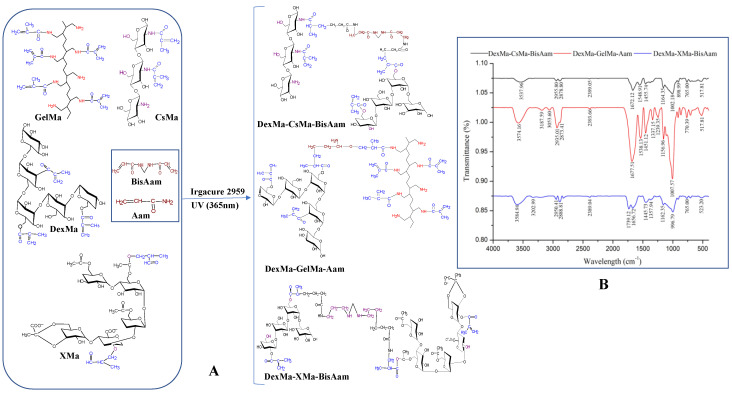
Schematic representation of the 3D network preparation (**A**) and FTIR data (**B**).

In this research, 3D networks based on dextran and chitosan/gelatin/xantan and synthetic polymers (polyacrylamide) were obtained by natural polymer methacrylation and photopolymerisation with acrylamide/acrylamide + N,N’methylenebis (acrylamide). The schematic reaction for hydrogel preparation and the FTIR data are presented in Figure 1.

Common vibrations in the infrared spectroscopy spectra were recorded at 1548.91 cm^−1^, absorptions corresponding to the carbonyl methacrylate groups (C=O stretching peak). The DexMa–XMa-based hydrogel spectrum presents small variations in the domain 1538.13–1656.72cm^−1^. Peaks at 1739.12 cm^−1^ and 1772.12 cm^−1^ are attributed to BisAam and at 1677.51cm^−1^ to Aam and the stretch vibration of the –COOH group, which confirmed the existence of the BisAam and Aam in the hydrogel’s composition [36].

The methacrylate group, identified in all the modified polymers, presents absorption at 1677.51 cm^−1^, 1239.35 cm^−1^, 898.99 cm^−1^, and 765.00 cm^−1^, which correspond to C=C stretching, the C=O stretch, CH_2_ rocking, out of phase, and the CH in-plane bend CH_2_ wag, the out-of-plane OH bend, the CH_2_ twist, and the CH_2_ group. Furthermore, the vibration of the -OH bond from the dextran methacrylate structure is present in all 3 spectra, at values between 1002.18 cm^−1^ and 1007.57 cm^−1^, being slightly attenuated by the C=C bond from the methacrylate xanthan structure. The peak value of 2955.80 cm^−1^ represents the C–H and C=O groups, stretches of amide groups from methacrylated chitosan (CsMa chains). The presence of pendant vinyl groups in the dextran–methacrylate was confirmed by the FT-IR bands at 1656.72 cm^−1^ (C=C) and 898.99 cm^−1^ (C=CH).

On the other hand, the bending vibration of the N-H group specific to amide (III) present at 1239.35 cm^−1^ is characteristic of the GelMa chain structure [37]. The spectrum of DexMa-GelMa-Aam has characteristic bands of GelMa at 1538.13 cm^−1^ (N–H bending vibration) and 1677.51 cm^−1^ (C=O stretching vibration), and a symmetric –NH_2_ stretching of the primary amide was found at 3187.59 cm^−1^.

High intensive absorption situated around 3574.16 cm^−1^ is attributed to acrylamide, and BisAam (3584 cm^−1^), representative of the N–H stretching vibrations, is specific to the amides structure [38]. The stretching peak of –OH at 3202.99 cm^−1^ in the DexMa-XMa-BisAam spectrum is attributed to the polymerization of methacrylic groups grafted onto Xanthane. In addition, there was a significant absorption at 1739.12 cm^−1^ (–C=O group) and a new peak at 2950.41 cm^−1^ caused by the stretching band of the C-H groups (ether group). Furthermore, the COO– group was responsible for the peak from 1445.73 cm^−1^ [39].

### 2.2. Hydrogel Morphology

**Figure 2:**
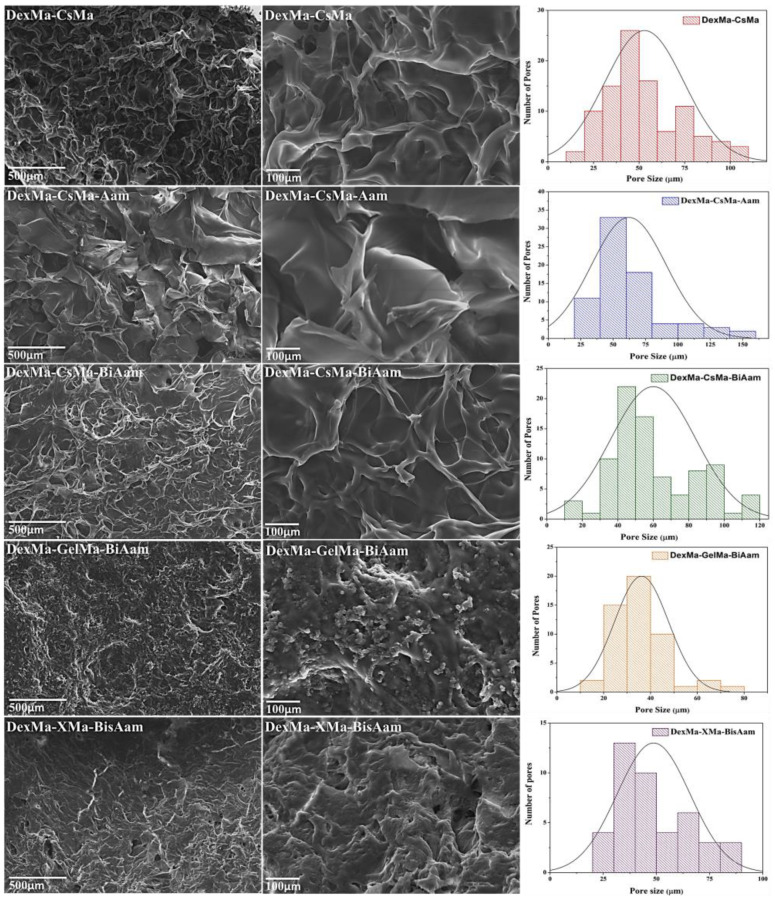
Scanning microscopy data for DexMa-CSMa, DexMa-CSMa-Aam, DexMa-CSMa-BisAam, DexMa-GelMa-BisAam, and DexMa-XMa-BisAam lyophilized hydrogels and the number and pore size; variations with hydrogel composition.

Scanning electron microscopy was used to obtain cross-section micrographs on lyophilized hydrogels, in order to correlate the hydrogels’ compositions and 3D architecture with their abilities to load and release drugs involved in skin cancer treatment and to evaluate their potential as drug delivery systems. The obtained micrographs revealed morphological differences between the analyzed samples, as shown in Figure 2 and Table 1.

### 2.3. Swelling Properties

Hydrogels are excellent materials in terms of their ability to absorb and retain large amounts of biological fluids, without disintegration or the release of structural components. This behavior makes them promising materials for a wide range of applications, such as regenerative medicine and drug delivery. The diffusion of the fluids through the network and deformation of the 3D structure in response to changes in the chemical/biochemical and physiological/pathological environments is a key issue in designing hydrogels for medical applications [40,41]. Under physiological conditions, the hydrogels’ swelling depends on the degree of ionization of the hydrophilic groups present alongside the polymeric chain, as well as the degree of crosslinking. Generally, the crosslinking density modifies the swelling behavior of the hydrogels, but the swelling properties are also influenced by specific interactions between swelling environment molecules (water, ions, biological molecules) and hydrophilic groups on the three-dimensional polymeric network (–OH, –COOH, –NH_2_, –NHCO–) [42]. The swelling behavior of the hydrogels and the amount of biological fluid in the hydrogel network modulate the transport properties of the hydrogel, and its biological and biomechanical activity.

**Figure 3:**
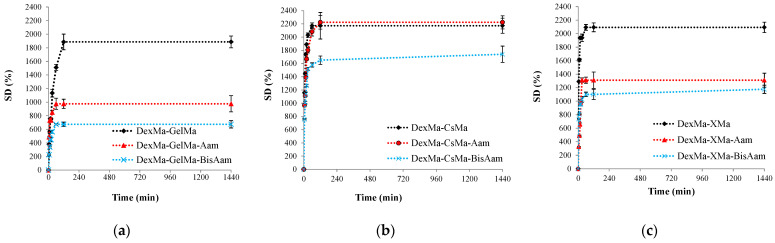
Kinetic swelling behavior of the hydrogels: (**a**) for GelMa based hydrogels, (**b**) for CsMa based hydrogels and (**c**) for XMa based hydrogels. Values are expressed as the mean of three independent experiments. Each value represents the mean ± standard error mean (*n* = 3).

The swelling properties were evaluated in a phosphate-buffered solution under simulated biological temperature conditions (37 °C), and the degree of swelling was kinetically monitored (Figure 3). The hydrogels’ swelling properties are attributed to both their macroporous structure and the hydrophilic functionality of the natural polymers [43,44].

All hydrogels reached the equilibrium swelling degree after about 60 min, and the maximum degree of swelling was strongly dependent on the combination of polymers from the materials and the presence of Aam and BisAam in the 3D network. Generally, both of the synthetic components reduced the degree of swelling, with Aam and BisAam acting as crosslinkers in the 3D network architecture. The DexMa-CsMa hydrogels reached higher values (SD > 2200%), confirming the presence of large intercommunicating pores, which favour the free movement of molecules inside the materials. Comparing the crosslinked hydrogels, DexMa-CsMa-BisAam, which has the highest porosity, presents superior swelling properties to DexMa-XMa-BisAam and DexMa-GelMa-BisAam, hydrogels with more collapsed 3D networks.

### 2.4. Bioadhesive Properties

**Figure 4:**
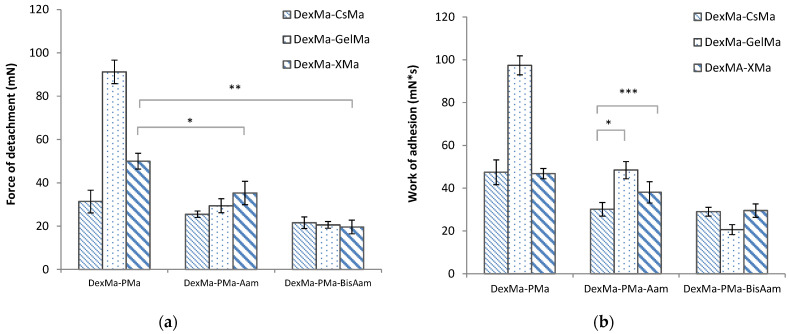
Bioadhesion properties of the hydrogels, determined as the detachment force (**a**) and work of adhesion (**b**). Values are expressed as the mean of six independent experiments. Each value represents the mean ± standard error mean (*n* = 6) (* *p* < 0.05, ** *p* < 0.01, *** *p* < 0.001).

In vitro bioadhesion studies on hydrogels were performed using a simulating membrane (cellulose membrane) and a TA.XT Plus texture analyzer. The force required for the detachment of the scaffolds from the biological membrane was recorded, and detachment forces with high values indicate improved bioadhesive properties. The force of detachment and work of adhesion of the materials decreased when Aam and bisAAm were added to the composition, as shown in Figure 4.

Generally, polymers containing polar functional groups (such as –COOH, –OH, –NH_2_, and –SO_4_) are considered highly bioadhesive and are combined in various formulations for drug delivery and tissue engineering [45]. Polymer characteristics, such as the chain length and spatial conformation, the charge and degree of hydration, the degree of crosslinking, or graftings, influence the bioadhesive properties and the biointeractions with tissues [46,47]. Gelatin is a collagen-derived protein with a large number of COOH, OH, and NH_2_ functional groups that are able to interact with cell membranes and glycoprotein chains, as well as with various tissues and biological environments. Moreover, gelatin’s structure includes the arginine-glycine-aspartic acid peptide, a bioactive sequence that facilitates cellular adhesion and proliferation [48]. Dextran’s bioadhesion properties can be controlled by oxidation, methacrylation, and grafting, or by combining the polysaccharide with proteins, including gelatin [49]. The gelatin- and dextran-based hydrogels presented increased values for the detachment force and the work of adhesion, three times and double those of the DexMa-CsMa and DexMa-XMa hydrogels, respectively, as a result of the synergic bioadhesive characteristics of native dextran and gelatin. However, all the polymer combinations studied presented values for the two parameters that recommend them for applications that require bioadhesive properties.

The bioadhesive polymers interact with biological tissues following a three-step phenomenon, namely: the wetting and swelling of the bioadhesive network, the interpenetration of polymer chains from hydrogels with tissue biomacromolecules, and, finally, the formation of chemical bonds. Generally, polymers with neutral and low charges physically interact through diffusion and interpenetration, followed by the physical entanglement of the polymer with the biological component. The extent of the bioadhesion depends on the free polymeric chains available for interlocking and crosslinking [50]. The presence of Aam and BisAam in the 3D networks forms tight crosslinked architectures; collapse or relaxation phenomena in the crosslinked hydrogels control the polymeric side chains that are available for entanglement with biological membranes and tissues, and therefore control the bioadhesion [51].

### 2.5. In Vitro Drug Release

**Figure 5:**
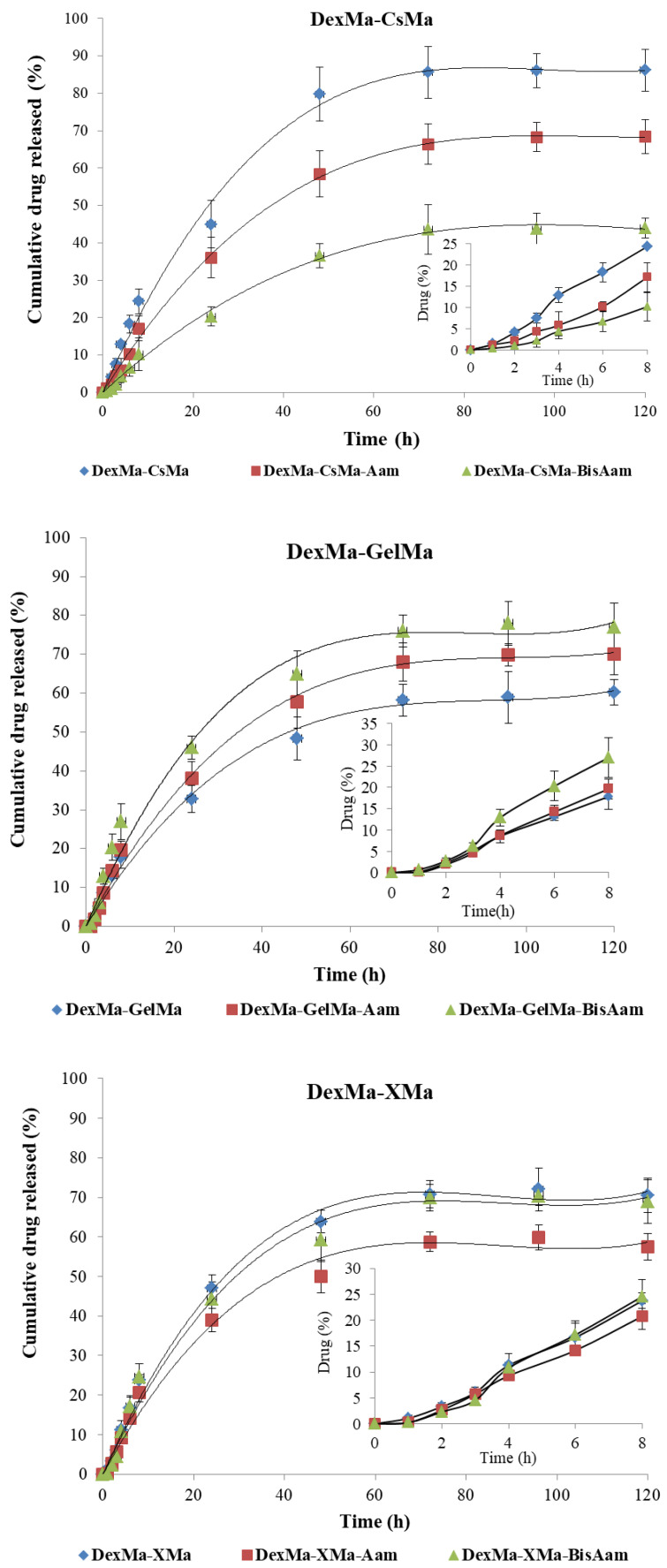
Kinetic release of Doxorubicine from hydrogels with various compositions. Values are expressed as the mean of three independent experiments. Each value represents the mean ± standard error mean (*n* = 3).

DOX loading into the hydrogels was performed using an alcoholic solution, with the aim of preserving their morphological structures and ensuring maximum drug loading (100% loading efficiency was considered). The in vitro release of Doxorubicin (Dox) from the hydrogels was performed in PBS (pH = 7.2, 0.01 M) at 37 °C, simulating the physiological environment. The cumulative release of the drug was plotted against time (Figure 5) in order to obtain the drug-release profiles and analyze the release mechanism. Doxorubicin (Dox), an anti-tumor agent that prevents DNA replication, affects the activity of topoisomerase II and cell functioning by binding to the cell membrane. It is recommended by the FDA for use in the treatment of a variety of cancers: acute lymphoblastic leukemia, soft tissue and bone sarcomas, breast carcinoma, ovarian carcinoma, thyroid and gastric carcinoma, Hodgkin’s disease, transitional cell bladder carcinoma, etc. Dox was first isolated from *S. peucetius var. caesius*; it is an anthracycline drug, with an amphiphilic molecule comprising the water-insoluble adriamycinone and a basic, reducing, water-soluble, amino-sugar functional group (daunosamine) [52]. It is well known that the systemic administration of Dox causes severe side effects, mainly because of its unselective toxicity on non-cancerous cells; these include DNA alterations caused by the presence of adriamycinone, which leads to the reduction or stopping of the cells’ growth. The principal side effects of the usage of Dox include nausea, vomiting, arrhythmia produced immediately after administration, and cardiotoxicity due to increased oxidative stress [53,54]. To limit these drawbacks, the local delivery of Dox from hydrogels is preferred in the treatment of skin cancer; this local mode of administration reduces the side effects and, moreover, the therapeutic effects are enhanced.

The release profiles of the hydrogels based on DexMa-CsMa indicated a steady, controlled release of the maximum concentration of the drug for at least 50 h. However, in the case of hydrogels with Aam and Aam-BisAam, equilibrium in the drug concentration was reached between the hydrogel and the environment at smaller amounts: 0.856 mg for DexMa-CsMa, 0.642 mg for DexMa-CsMa-Aam-BisAam, and 0.420 mg for DexMa-CsMa- Aam-BisAam. This can be explained by the interactions between Dox and the Aam/Aam-BisAam fragments from the polymeric networks. In the hydrogels, DexMa-GelMa and DexMa-XMa, this effect is mitigated.

In its hydrochloride state, as it was used in the performed tests, the Dox molecule is readily soluble in water, slightly soluble in normal saline and PBS, and sparingly soluble in alcohol. The drug loading and release capacity depend on the networks’ constitutive polymers, their crosslinking and hydrogel morphology, the drug molecular volume, and drug–hydrogel interactions [55]. Ionic interactions between the COOH and NH_2_ groups from Dox and similar groups from the natural polymer, as well as the hydrogen bonds with Aam and BisAam, are evident in all hydrogels. The significant differences in the hydrogel–drug interactions are more strongly correlated with the type of natural polymer than with the presence of a synthetic component.

As is generally known, drug release from hydrogels is governed by several mechanisms: swelling, diffusion, network relaxation, or erosion. Often, all of them are present to varying degrees, depending on the polymer type, network crosslinking degree and hydrophylicity, and the drug’s structure, charge, and molecular volume, as well as its interaction with the polymeric matrix [56]. In order to study the drug release kinetics and mechanism, two mathematical models were used to fit the release data: the Higuchi and Korsmeyer–Peppas models [35,57].

The Higuchi equation is based on the first law of diffusion (Fick’s law) and is used in relation to various porous systems and the release kinetics of slightly water-soluble species that are encapsulated into matrices (Equation (1)):

(1) $$M\left(t\right)=k^{n}\times t^{1/2}$$

where *k_H_* is the release constant of Higuchi (expressed in mg × min^−1/2^) and *Mt* is the drug concentration at the time *t*.

The Korsmeyer–Peppas model was developed to describe drug release from polymeric systems (Equation (2)):

(2) $$\frac{M_{t}}{M_{\infty }}=k\times t^{n}$$

where *M_t_* is the amount of the drug released at the time *t*, *M_∞_* is the amount of the drug at equilibrium, *k* is the release rate constant, which characterizes the drug–matrix system, and *n* is the exponent that indicates the drug release mechanism.

A value of the exponent n equal to 0.5 indicates a Fickian diffusional mechanism of the drug from the inside of the hydrogel, and a value between 0.5 and 1 for the exponent of release indicates non-Fickian diffusion (i.e., the drug delivery is dictated by the swelling and the polymeric chain relaxation). The values for the correlation coefficients of the applied mathematical models, the release rate constant (*k*) and the release exponent (*n*), are presented in Table 2.

The obtained results suggest that, for all hydrogels, the release mechanism followed Korsmeyer–Peppas kinetics, with the correlation coefficients registering values over 0.99 [58]. In addition to these data, the release rate constant (*k*) and release exponent (*n*) were calculated, in order to determine the mechanism of drug release from hydrogels. The values obtained for the exponent n suggested a close-to-Fickian transport (*n* close to 0.5), which indicated that the drug is released from the hydrogels by diffusion for all the produced hydrogels, with a small contribution made by the hydrogel networks’ relaxation. All of these results indicate that Dox release can be controlled by selecting the natural polymers and the synthetic component in the hydrogel’s architecture.

### 2.6. In Vitro Cytotoxicity Studies

**Figure 6:**
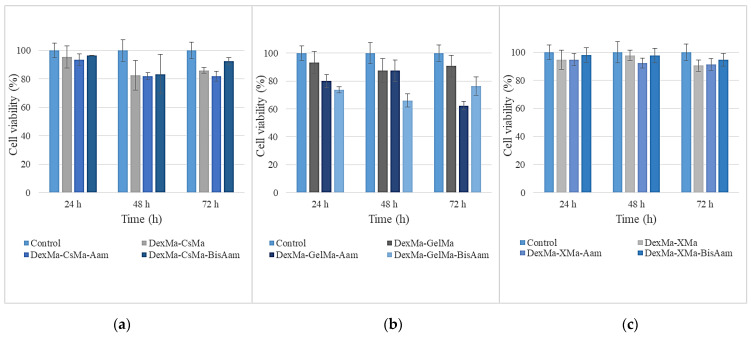
Cell viability after 24 h, 48 h, and 72 h of cell culturing for CsMa hydrogels (**a**), GelMa hydrogels (**b**) and XMa hydrogels (**c**). Values are expressed as the mean of three independent experiments. Each value represents the mean ± standard error mean (*n* = 3).

The cytotoxicity studies of the new synthetized hydrogels were performed on an A431 cell line, the results being shown in Figure 6. It can be observed that, for DexMa-CsMa-BisAam and DexMa-XMa-BisAam, the cell viabilities are well above the reference limits of ISO10993 for non-cytotoxic materials. Meanwhile, for DexMa-GelMa-Aam, the cell viabilities are around 70% compared to the control; this may be caused by the crosslinking mechanism that allows the synthetic polymer to be released into the culture medium, thus affecting the division ability of the cells. Studies have indicated that acrylamide induces cytotoxic as well as genotoxic effects through oxidative stress, which eventually lead to decreased cell viability through apoptosis and DNA damage, even in cancer cells [59].

**Figure 7:**
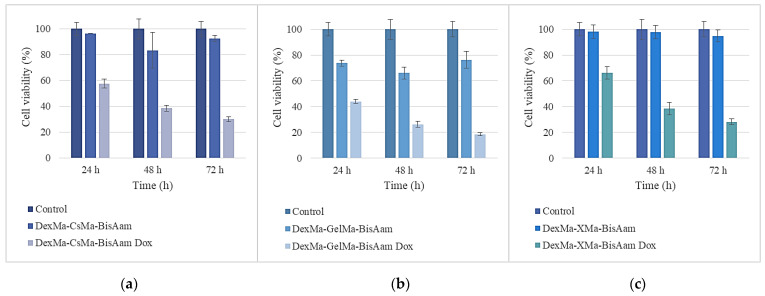
Cell viability of the DexMa-CSMa-BisAam (**a**), DexMa-GelMa-BisAam (**b**), and DexMa-XMa-BisAam (**c**) hydrogels after 24 h, 48 h, and 72 h of cell culturing. Values are expressed as the mean of three independent experiments. Each value represents the mean ± standard error mean (*n* = 3).

Given the purpose of our research and the fact that the obtained hydrogels are being considered for applications related to skin cells, cytotoxicity testing and drug release in the cellular environment were continued for those hydrogels that had adequate manipulation properties and behaviors under the culture conditions. Therefore, the following methacrylated polysaccharides, in combination with synthetic polymer-based hydrogels, were chosen to be studied in more detail regarding their cytotoxicity and drug-release abilities: methacrylated dextran-methacrylated chitosan-bisacrylamide (DexMa-CsMa-BisAam); methacrylated dextran-methacrylated gelatin-acrylamide (DexMa-GelMa-BisAam); and methacrylated dextran-methacrylated xanthan-bis-acrylamide (DexMa-X-Ma-BisAam). The MTT test results for this set of hydrogels are shown in Figure 7.

Considerable differences can be observed in cell viability between the simple and doxorubicin-loaded hydrogels. The effects of the released doxorubicin on cell viability are strictly correlated with the drug-release profile presented in Section 2.5. The mechanism through which doxorubicin affects cell division and the reason why it is used as an antitumor drug relate to its insertion between DNA base pairs, inhibiting DNA synthesis. It possesses topoisomerase-II-inhibition properties and activity that determines the accumulation of oxygen-reactive species at levels that induce cellular damage [60]. These known effects of doxorubicin are not limited to cancer cells, but also apply to normal cells, especially cardiac cells [19,61]. This is why this research aims to achieve the local release of the drug, thus avoiding systemic effects.

**Figure 8:**
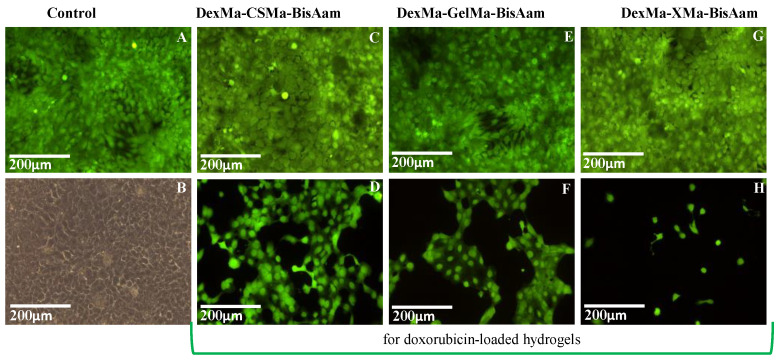
Cell morphology after four days of exposure to the DexMa-CSMa-BisAam (**C**,**D**), DexMa-GelMa-BisAam (**E**,**F**), and DexMa-XMa-BisAam (**G**,**H**) hydrogels compared to control (**A**,**B**). Images (**D**,**F**,**H**) present the effects of doxorubicin-loaded hydrogels on cell morphology and viability (Calcein AM staining).

After 24 h of exposure to doxorubicin-loaded hydrogels, a cell viability of between 43 and 66% was determined, as a consequence of drug release, as compared to the control; the gelatin-based hydrogel had the lowest percentage of viable cells. Moreover, 48 h after doxorubicin exposure, the trend was maintained and cell viability was reduced to 26–38%; then, 72 h later, the values were found to be in the 18–29% interval. Differences in cell viability between the simple and drug-loaded hydrogels were also observed using Calcein AM for cell staining. This also allowed us to observe cell morphology; it can be seen that, in this regard, there are no differences between the control cells and the hydrogel-exposed cells. For doxorubicin-loaded hydrogels, the MTT results are in clear correlation with the fluorescence microscopy aspect (Figure 8D,F,H), as the drug has affected the division ability of the cells.

**Figure 9:**
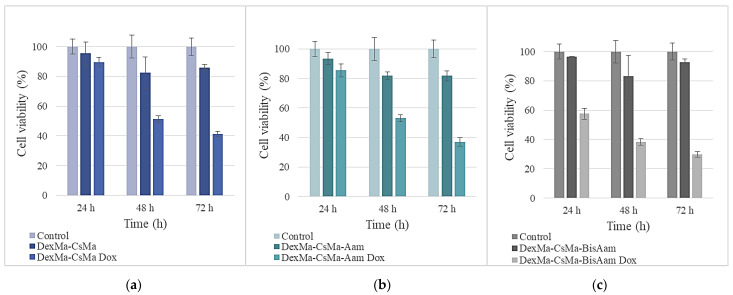
Cell viability for the Dex-CsMa (**a**), Dex-CsMa-Aam (**b**), and Dex-CsMa-BisAam (**c**) hydrogels after 24 h, 48 h, and 72 h cell of cell culturing. Values are expressed as the mean of three independent experiments. Each value represents the mean ± standard error mean (*n* = 3).

Considering the promising results obtained for the DexMa-CsMa-BisAam hydrogels, DexMa-CsMa-based hydrogels were chosen for further detailed study, as they proved to present adequate cytotoxicity results, manipulation properties, and drug release profiles. Chitosan’s properties make this polysaccharide a very important candidate in drug delivery research, as it proved to be cell permeable and with adequate mucoadhesive properties; meanwhile, the positive charge of its amino group was shown to be attracted by the tumor cell membrane, which has a higher negative charge compared to non-tumor cells [62,63]. To explore chitosan’s potential, the DexMa-CsMa and DexMa-CsMa-Aam hydrogels were synthetized and tested under the same conditions as previously described. The MTT results can be seen in Figure 9, showing similar viability values for methacrylated polysaccharide-based hydrogels either with or without the synthetic component. Bisacrylamide-containing hydrogels showed the highest cell viability, which might be determined by the crosslinking role of this molecule, assuring an improved interaction between the hydrogel’s components. A slower doxorubicin release profile of these hydrogels is correlated with higher cell viability in the first 72 h compared to the gelatin- or xanthan-based hydrogels.

**Figure 10:**
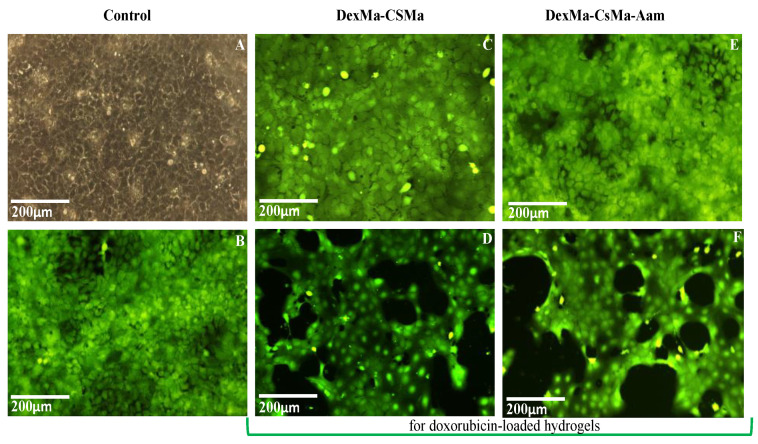
Cell morphology after four days of exposure to the DexMa-CSMa (**C**,**D**) and DexMa-CSMa-Aam (**E**,**F**) hydrogels compared to the control (**A**,**B**). Images (**D**,**F**) present the effects of doxorubicin-loaded hydrogels on cell morphology and viability (Calcein AM staining).

Cell morphology and viability were also assessed for these chitosan-based hydrogel variations, and no differences in cell morphology were observed between the control and hydrogel-exposed cells (Figure 10).

Doxorubicin-exposed cells present modifications of the morphology in the form of cytoplasm shrinkage and the formation of apoptotic bodies or membrane blabbing, which finally leads to apoptosis [34]. Meanwhile, the influence of the drug on the cell division process can be observed in the fluorescence microscopy images as empty areas on the culture plate well, as compared to non-exposed cells. Further studies will be performed in order to elucidate the effect of Dox in A431 tumoral cells (with biomarkers such as AMPK, p53, and Bcl-2, which have been identified as important for apoptosis induction by doxorubicin [64]) and to correlate the concentration of the drug loaded in the hydrogels with its biological effects.

## 3. Conclusions

The aim of the present work was to obtain drug-loaded hydrogels based on combinations of Dextran and chitosan/gelatin/xantan, crosslinked with a synthetic polymer (derived from acrylamide and N,N’-methylenebis(acrylamide)) to improve the release profile of Doxorubicin from an immediate release type to a sustained and controlled release type, as is recommended for topical delivery in skin cancer therapy. In order to prepare versatile hydrogels with good manipulation characteristics and convenient drug delivery profiles, dextran, gelatin, chitosan, and xanthan were used as methacrylated derivatives, moieties that are able to polymerize with synthetic monomers and to produce 3D hydrophilic networks. FT-IR analysis confirmed the hydrogels’ network structure, while SEM microscopy analysis confirmed the porosity of the materials’ hydrogels and the interconnected and dimensional variable macropores, which offer the possibility of retaining fluids by diffusion in order to modulate the drug release profile and to maintain adequate drug concentration at the application site.

All of the hydrogels reached a high equilibrium swelling degree in a simulated biological fluid, and the maximum swelling degree was obtained for the dextran-chitosan-based hydrogels; the data correlate the swelling properties with the hydrogels’ morphology (i.e., porosity and pore distribution). The hydrogels are bioadhesive on a simulated biological membrane, and the values for the force of detachment and work of adhesion are superior for the hydrogels based on dextran and gelatin. Doxorubicin was loaded into the hydrogels and released by diffusion for all the produced hydrogels, with a small contribution made by the hydrogel network’s relaxation. Doxorubicin-loaded hydrogels are effective on malignant keratinocyte cells, the data indicating the drug’s effects on cell division and apoptosis. However, the study should be taken further by evaluating the drug’s interactions with hydrogels and the correlations between the loaded drug and its biological effects when released.

## 4. Materials and Methods

### 4.1. Materials

Natural polymers obtained from Sigma-Aldrich, Darmstadt, Germany and methacrylated using the protocol described in [65] were used to obtain the hydrogels: high-molecular-weight chitosan (CsMa, Mw = 310.000–375.000 Da, 21.2% degree of methacrylation); gelatin (GelMa, from porcine skin, Mw = 100,000 Da, 62.4% degree of methacrylation); dextran from *Leuconostoc* spp. (DexMa, Mw = 450.000–650.000 Da, 15.1% degree of methacrylation); and xanthan from *Xanthomonas campestris* (XMa, Mw = 458,000 Da, 9.5% degree of methacrylation). The monomers (acrylamide -Aam and N,N’-methylenebis (acrylamide), bisAam)) and the photoinitiator (Irgacure 2959 (2-Hydroxy-4′-(2-hydroxyethoxy)-2-methylpropiophenone)) were purchased from Sigma-Aldrich, Darmstadt, Germany. Absolute ethyl alcohol, the dialysis membrane (Mw = 12,000–14,000 Da), isopropanol, Dulbecco’s Modified Eagle’s Medium/Nutrient Mixture F-12 Ham, Hanks’ Balanced Salt Solution, MTT (3-(4,5-dimethylthiazol-2-yl)-2,5-diphenyltetrazolium), and PBS (phosphate-buffered saline, pH = 7.2) were provided by Sigma-Aldrich, Darmstadt, Germany. Other essential elements used in the study are the antitumor drug doxorubicin hydrochloride (DOX, provided by Sigma-Aldrich, Darmstadt, Germany) and epidermal carcinoma cells A431 (acquired from the European Collection of Cell Cultures (ECACC), Salisbury, United Kingdom).

### 4.2. Synthesis of Hydrogels

Three combinations of methacrylated polymers were selected for the hydrogel preparation: dextran (DexMa)—chitosan (CsMa), dextran (DexMa)—gelatin (GelMa) and dextran (DexMa)—xanthan (XMa). The hydrogels’ composition is presented in Table 3.

The hydrogels based on dextran and chitosan were prepared as follows. Methacrylated dextran solutions (3% (*w*/*v*) in a phosphate buffered solution, 0.01 M, pH 7.2, and methacrylated chitosan (3% (*w*/*v*) in a phosphate buffered solution, 0.01 M, pH 7.2 were continuously stirred (300 rpm) until a homogenous mixture was obtained. The photoinitiating agent (Irgacure, 2% (*w*/*v*)) and calculated amounts of acrylamide (Aam, 10% (wt/wt), reported to polymers)/acrylamide (Aam) and N, N′-methylene-bisacrylamide (BisAam) (Aam, 8% (wt/wt), BisAam 2% (wt/wt), reported to polymers) were added and homogenized. The obtained mixtures were poured in 24-well cell culture plates (500 µL per well) and exposed to UV light (λ = 365 nm, 5 min). The obtained hydrogels were freeze-dried. Freeze-drying was carried out with a FreeZone benchtop freeze-drier (Labconco, Kansas City, MO, USA), and a cooling rate of 1 °C min^−1^ was used until the freezing temperature of −54 °C was obtained, with a vacuum of 41mTorr at 0 °C. Finally, the hydrogels were washed with a mixture of 90% absolute ethyl alcohol and 10% distilled water for the removal of residual products.

### 4.3. Hydrogel Characterisation

#### 4.3.1. Fourier Transform Infrared Spectroscopy (FTIR) and Scanning Electron Microscopy (SEM)

FTIR spectra were recorded on dried samples in KBr pellets (1% dried material) using a Bruker Vertex 70 spectrophotometer (Berlin, Germany) and scanned within the range of 400–4000 cm^−1^ in transmittance mode. The cross-section morphology of the lyophilized hydrogels was analyzed using a HITACHI SU 1510 scanning electron microscope (Hitachi SU-1510, Hitachi Company, Tokyo, Japan) in a Secondary Electron (SE) system. For sample preparation, a transversal sectioning of the dry material was conducted using a sharp scalpel; then, the exposed sections were mounted on an aluminum stub and fixed. All the analyzed samples were coated with a 7 nm gold layer using a Cressington 108 Sputter Coater. The SEM images were analyzed using ImageJ software.

#### 4.3.2. Swelling Properties

The swelling property tests were performed in simulated physiological conditions. The hydrogels were completely submerged in a QIA quick VR spin column 50, Ø 10mm, connected to a 1 mL syringe containing phosphate-buffered solution (PBS), with pH = 7.2 and 0.01 M; they were then incubated at 37 °C. The swelling degree values were calculated using the following equation:

(3) $$SD\left(\%\right)=\frac{w_{t}-w_{o}}{w_{o}}\times 100$$

where *w_t_* represents the weight of the sample at time *t* and *w_o_* is the initial weight of the dried sample. The experiments were performed in triplicate, and the results are expressed as the mean ± standard deviation.

#### 4.3.3. Bioadhesive Characteristics

A TA.XT Plus^®^ texture analyzer (Stable Micro Systems, UK) and a simulated biological membrane (cellulose membrane—4 cm^2^, 12,000 Da, from a dialysis tubing system, pre-boiled and cooled at room temperature) were used to measure the bioadhesion of the hydrogels [66,67]. The tests on the cellulose membrane have been confirmed to correlate well with those obtained from animal mucosa tissues [68]. The membrane was fixed in the static device and 200 µL of phosphate-buffered solution (pH 7.4 and 0.01 M) was added for the simulation of the physiological environment. After that, the holding device was placed in a controlled temperature system (distilled water was heated to 37 °C and stirred at 150 rpm). Pieces of the dried hydrogels (ϕ = 8 mm) were attached to the moving device (a cylindrical graphite probe (P/8), with an 8 mm diameter) and then lowered with a pre-determined speed of 1 mm/s until making contact with simulated membrane. The contact was maintained for 30 s (contact force of 9.80665 mN) and then the upper part of the texture analyzer was lifted at a speed of 0.1 mm/s until separation was achieved. Using the Texture Exponent software, the data were collected and analyzed, and parameters such as the maximum detachment force and the work of adhesion were calculated based on the force–time plots. Six replicates were measured and the results were averaged.

#### 4.3.4. Drug-Loading and Drug-Release Studies

The dried hydrogel disks were immersed in an alcoholic solution of DOX (0.5 mg/mL, in ethylic alcohol) and kept for 24 h in the dark and at room temperature. The solvent was removed by convective drying at 25 °C. The release experiments were carried out by immersing the drug-incorporating hydrogels into a dialysis membrane with 4 mL solution of PBS (pH = 7.4). This volume was calculated according to the mass of each sample. Finally, the hydrogel-dialysis membrane–PBS systems were immersed in 15 mL of PBS and incubated at 37 °C. Then, 1 mL of the release medium was withdrawn periodically and replaced with 1 mL of fresh PBS. The amount of Dox in the release medium was measured spectrophotometrically at a wavelength of 485 nm and the cumulative release data were obtained using a calibration curve for Doxorubicin.

#### 4.3.5. In vitro Cytotoxicity Studies

The cytotoxicity of the obtained hydrogels was tested according to ISO10993 recommendations in order to determine their effect on the A431 epidermal cell line. The cells were cultured in a 75 cm^2^ flask, in Dulbecco’s Modified Eagle’s Medium/Nutrient Mixture F-12 Ham (DMEM F12/Ham) completed with 10% FBS and 1% antibiotics, with the medium changed every other day. Upon the formation of a monolayer, the adherent cells were dissociated by trypsinization, counted, and tested for viability with trypan blue. After this, 48-well plates were used for cell distribution, with 1 × 10^4^ cells/well, and cultured for 24 h. The next day, fresh medium was added to each well and the hydrogels were distributed in triplicate to each well, except for those left as controls. For this stage of testing, simple and doxorubicin-loaded hydrogels were used. The MTT test was performed after 24, 48, and 72 h of direct hydrogel contact with the cells, according to a procedure described previously. Briefly, the hydrogels were carefully removed from the wells and the medium was replaced with fresh DMEM without FBS, with 5 mg/mL MTT, and left in incubation conditions for 3 h, after which the formed formazan crystals were solubilized with isopropanol and the absorbencies were determined using an UV/Vis plate reader (Tecan Sunrise Plate Reader, Tecan Trading AG, Männedorf, Switzerland) at 570 nm. The cell viability was calculated according to Equation (4):

(4) $$\mathrm{Cell}\mathrm{viability}(\%)=\frac{Abs\;sample}{Abs\;control}\times 100$$

where *Abs sample* represents the absorbance in the well with the hydrogel, while Abs control is the absorbance in the well with cells only.

##### Hydrogel Preparation for Cytotoxicity Testing

For the evaluation of the hydrogels’ effects on the tested cells, the direct contact method was used. For this, the hydrogels were prepared by cutting them into equal squares, and materials of the same weight were used. All the hydrogels were sterilized in 70% sterile filtered ethanol, washed 3 times in HBSS for complete ethanol removal, and then left to dry in sterile conditions. After being completely dried, the hydrogels were rehydrated in DMEM F12/Ham for 24 h. For those materials loaded with doxorubicin, the rehydration step was undertaken in doxorubicin containing DMEM F12/Ham at a concentration of 5 µg/mL.

##### Cell Morphology Evaluation by Fluorescence Microscopy

Along with the cytotoxicity testing of the hydrogels, cell viability and morphology were evaluated by fluorescence microscopy after 4 days of direct contact between the hydrogel and the cells. A Calcein AM solution (2 µM) was prepared in calcium and magnesium containing HBSS and used for cell staining; the cell analysis was performed using an inverted fluorescence Leica DMI3000 microscope (Leica Microsystems GmbH, Wetzlar, Germany) and compared with the control cells.

#### 4.3.6. Statistical Analysis

The results obtained were expressed as the mean ± standard deviation (SD) of the mean values for each experiment, made in triplicate. The bioadhesion tests required 6 replicates for the considerable reduction of method errors. Statistical analysis was performed by applying one-way ANOVA and Tukey post-hoc analysis. Values of the *p* coefficient (probability) lower than 0.05 were considered to be statistically significant.

## Figures and Tables

**Table 1:** Pore size variations with hydrogel composition.

DexMa-CsMa (µm)		DexMa-CsMa-Aam (µm)		DexMa-CsMa-BisAam (µm)		DexMa-GelMa-BisAam (µm)		DexMa-XMa-BisAam (µm)	
Min	Max	Min	Max	Min	Max	Min	Max	Min	Max
27.75 ± 4.17	80.95 ± 6.43	47.15 ± 3.49	143.10 ± 9.12	52.07 ± 6.88	131.46 ± 2.09	30.68 ± 5.04	46.15 ± 4.66	41.95 ± 11.17	88.96 ± 6.09

**Table 2:** Release rate constant (*k*) and release exponent (*n*).

Hydrogel	Correlation Coefficient (r2)		Release Rate Constant, *k* (h^−n^)	Release Exponent, *n*
	Higuchi	Korsmeyer–Peppas		
DexMa-CsMa	0.9812	0.9955	0.1403	0.5629
DexMa-CsMa-Aam	0.9799	0.9962	0.1612	0.5719
DexMa-CsMa-BisAam	0.9843	0.9987	0.1822	0.5685
DexMa-GelMa	0.9892	0.9904	0.1074	0.5270
DexMa-GelMa-Aam	0.9792	0.9973	0.0934	0.5374
DexMa-GelMa-BisAam	0.9765	0.9965	0.0962	0.5907
DexMa-XMa	0.9803	0.9944	0.0962	0.5507
DexMa-XMa-Aam	0.9789	0.9952	0.0943	0.5531
DexMa-XMa- BisAam	0.9806	0.9986	0.1019	0.5496

**Table 3:** Hydrogel composition.

Hydrogels	DexMa (%)	CsMa (%)	GelMa (%)	XMa (%)	Aam (%)	BisAam (%)
DexMa-CsMa	50	50	-	-	-	-
DexMa-CsMa-Aam	45	45	-	-	10	
DexMa-CsMa-BisAam	45	45	-	-	8	2
DexMa-GelMa	50	-	50	-	-	-
DexMa-GelMa-Aam	45	-	45	-	10	
DexMa-GelMa-BisAam	45	-	45	-	8	2
DexMa-XMa	50	-	-	50	-	-
DexMa-XMa-Aam	45	-	-	45	10	
DexMa-XMa-BisAam	45	-	-	45	8	2

## Data Availability

Not applicable.
